# Is High Milk Intake Good for Children’s Health? A National Population-Based Observational Cohort Study

**DOI:** 10.3390/nu13103494

**Published:** 2021-10-02

**Authors:** Yoowon Kwon, Seung Won Lee, Young Sun Cho, Su Jin Jeong, Man Yong Han

**Affiliations:** 1 CHA Bundang Medical Center, Departments of Pediatrics, CHA University School of Medicine, Seongnam 13496, Korea; youyisi68@gmail.com (Y.K.); chocoloys@gmail.com (Y.S.C.); 2 Department of Data Science, Sejong University College of Software Convergence, Seoul 05006, Korea; lsw2920@gmail.com

**Keywords:** milk, body weight, nutritional state, iron deficiency anemia, children

## Abstract

Milk is widely considered as a beneficial product for growing children. This study was designed to describe the milk consumption status of Korean children aged 30–36 months and to investigate its association with the risk of obesity and iron deficiency anemia (IDA). This nationwide administrative study used data from the Korean national health insurance system and child health screening examinations for children born in 2008 and 2009. In total, 425,583 children were included, and they were divided into three groups based on daily milk consumption: low milk group (do not drink or drink <200 mL milk per day, *n* = 139,659), reference group (drink 200–499 mL milk per day, *n* = 255,670), and high milk group (drink ≥500 mL milk per day, *n* = 30,254). After adjusting variable confounding factors, the consumption of a large amount of milk of ≥500 mL per day at the age of 30–36 months was associated with an increased risk of obesity at the age of 42–72 months and IDA after the age of 30 months. These results may provide partial evidence for dietary guidelines for milk consumption in children that are conducive to health.

## 1. Introduction

Appropriate linear growth and weight gain are the most crucial issues among healthy children and are related to several factors, such as nutritional status, physical development, calorie intake from diverse types and amounts of food, and level of physical activity [1]. In particular, milk is widely considered as a beneficial product for growing children because it is a complete source of energy and is the richest and the most inexpensive source of high nutritional quality protein, calcium, phosphorus, and vitamin A [2,3]. Consequently, nutritional guidelines in most countries recommend daily milk consumption as a component of a healthy diet [4]. For instance, in the United States (US), the national dietary guidelines recommend amounts of dairy in 2, 2-1/2, and 3 cup equivalents per day for children aged 2–3, 4–8, and 9–18 years, respectively [5]. In China, the Chinese Dietary Guidelines 2016 suggest that school-age children should drink 300 mL of milk per day [1,6]. In Korea, the 2020 Dietary Reference Intakes for Koreans recommends two cups (400 mL) of milk for adolescents and one cup (200 mL) of milk for adults per day [7].

As growth issues such as obesity and stunting have become more of a concern, extensive research has been conducted on milk intake and height or body weight [1], and the majority of these studies have agreed that milk contributes positive benefits to growth in height for children [8,9,10,11,12,13,14]. However, the association between milk and body weight or body mass index (BMI) remains under debate due to inconsistent findings [15,16,17,18,19,20,21,22,23].

In fact, the basis for the dietary guidelines for milk is not sufficient, and data on Korean children’s actual daily milk consumption have remained scarce. In Korea, after the period of bottle-feeding, the amount of milk consumed significantly decreases, or conversely, if the time to stop bottle-feeding has elapsed and the child still depends on the bottle, the majority of calories per day tend to be consumed through milk. Several observational studies have suggested associations between prolonged bottle-feeding and excessive milk intake [24,25,26]. In this case, an imbalance of nutrients, especially insufficient intake of nutrients that milk does not contain or lacks, may occur. For instance, the finding that consumption of cow’s milk by infants and toddlers has adverse effects on iron stores has been documented in many studies [27]. The prevalence of iron deficiency anemia (IDA), which can significantly affect neurodevelopment, has been reported to increase in children aged 1 to 3 years, who have high iron requirements due to rapid growth [28,29]. When a direct relationship exists between milk intake and the growth or nutritional status of children, more specific recommendations on milk intake are essential.

This study was designed to describe the milk consumption status of Korean children aged 30–36 months (generally, after the cessation of bottle-feeding in Korea) and to investigate its association with the BMI z-scores of children aged 42–72 months. Moreover, by analyzing the relationship between milk intake and the risk of IDA, we attempt to obtain more information with regard to milk intake and nutritional status. We anticipate that the findings of this study would help set an appropriate range for daily milk consumption conducive to the health of Korean children.

## 2. Materials and Methods

### 2.1. Database: National Investigation of Birth Cohort in Korea Study 2008 (NICKs-2008)

The NICKs-2008, which integrates data from the Korean National Health Insurance System (NHIS) and the National Health Screening Program for Infants and Children (NHSPIC), consists of children born in 2008 (*n* = 469,248) and 2009 (*n* = 448,459) in the Republic of Korea.

The NHIS accumulates nationwide healthcare data that contain health records, including sociodemographic variables (age, sex, residential area, and income), healthcare utilization (International Classification of Diseases, 10th revision (ICD-10 codes)), procedure codes, drug classification codes, length of stay, and treatment costs for the entire nation. The NHSPIC conducts seven surveys among children, from the age of 4 to 72 months, comprising a general health questionnaire, developmental screening, anthropometric examination, physical examination, assessment of oral health, and age-specific anticipatory guidance. Detailed information on this study has been provided elsewhere [30].

### 2.2. Study Setting

This study was designed using the data from the NICKs-2008 database, which consisted of 917,707 subjects—all children born in 2008 and 2009 in Korea. Among the children who underwent the 4th round of NHSPIC, from the age of 30 to 36 months, children whose weight and height were properly recorded were included in the analysis (*n* = 425,583), excluding those who met the exclusion criteria. Exposure was defined as the consumption of milk per day, which was recorded from the results of the 4th round of the NHSPIC program. The primary outcome was the association between milk consumption and obesity at the age from 42 to 72 months. The association between milk consumption and IDA was also analyzed. The study protocol was reviewed and approved by the Institutional Review Board of the Korea National Institute for Bioethics Policy (P01-201603-21-005).

### 2.3. Study Population

**Figure 1:**
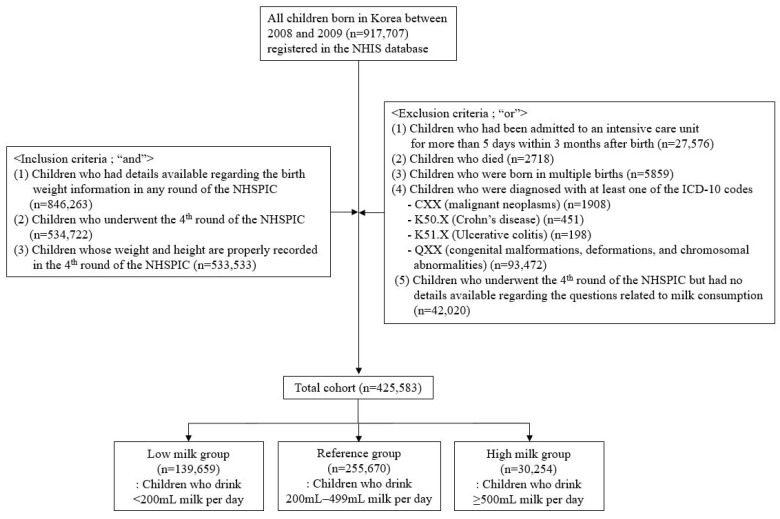
Enrollment diagram and grouping of the study population.

All children who were born during 2008–2009 and who participated in the health screening program were identified (*n* = 917,707). We included children (1) who had details available regarding birth weight information in any round of the NHSPIC, (2) who underwent the 4th round of NHSPIC, and (3) whose weight and height were properly recorded in the 4th round of NHSPIC (*n* = 533,533). Children that were excluded included those (1) who had been admitted to an intensive care unit for more than 5 days within 3 months after birth, (2) who died, (3) who were born in multiple births, (4) who were diagnosed with at least one of the ICD-10 codes (CXX (malignant neoplasms), K50.X (Crohn’s disease), K51.X (ulcerative colitis), and QXX (congenital malformations, deformations, and chromosomal abnormalities)), or (5) who underwent the 4th round of NHSPIC but had no details available regarding the questions related to milk consumption. In total, 425,583 children were included in this study, and they were divided into three groups based on daily milk consumption (Figure 1).

### 2.4. Exposure: Daily Milk Consumption

The exposure of interest was consumption of milk per day in children aged 30–36 months, which was recorded from the results of the 4th round of the NHSPIC program. Parents answered the following question: “How much milk does your child drink per day?” with the following possible answers: (1) Do not drink, (2) <200 mL, (3) 200–499 mL, (4) 500–999 mL, and (5) >1000 mL. Based on the answers, the children were divided into the following three groups: low milk group—children who do not drink milk or drink <200 mL milk per day (*n* = 139,659), reference group—children who drink 200–499 mL milk per day (*n* = 255,670), and high milk group—children who drink ≥500 mL milk per day (*n* = 30,254). In this study, milk refers to any plain whole, low-fat, and skim cow’s milk, not other types of milk such as goat milk or soy milk.

### 2.5. Outcomes: Obesity and IDA

The primary outcome was the association between milk consumption and obesity at the age of 42 to 72 months. The early adiposity rebound (AR) period (the age of 4–7 years), known as the risk period for obesity, has been determined to have a particularly important relationship with the prevalence of childhood obesity [31,32]. Obesity was defined as BMI z-score of ≥1.64 (95th percentile) [33,34] based on the BMI recorded at the last round of the 5th–7th NHSPIC [35,36,37]. BMI was calculated as weight in kilograms divided by height in meters squared.

The additional outcome was the association between milk consumption and IDA. We defined IDA using at least one diagnosis of the ICD-10 code D50.8 (other IDAs) or D50.9 (IDA, unspecified), along with prescription of iron supplements using drug classification codes 322 (Supplementary Table S1). Prescription at least once from the 4th round of NHSPIC to December 2017 was included.

### 2.6. Statistical Analysis

Data were analyzed using descriptive statistics and presented as counts (percentages) or mean (SD) values. For the analysis of the risk of obesity and IDA based on the milk consumption status in children aged 30–36 months, the relative risk (RR) and 95% confidence intervals (CIs) were estimated using generalized estimated equations, with the GENMOD procedure with log link function and all interactions of these variables. All RRs were presented with 95% CIs, and adjusted RRs (aRRs) in the multivariable analysis considered sex, birth residence, income quintile, birth year, prematurity, birth weight (continuous variable), type of milk feeding from age 4 to 6 months, and amounts of sugar-containing beverages (SCBs) from age 18 to 24 months. Probability (*p*) values of ≤0.05 were considered to be statistically significant. All statistical analyses were conducted using SAS version 9.4 (SAS Institute Inc., Cary, NC, USA).

## 3. Results

### 3.1. Daily Milk Consumption during Childhood in Korea and Comparison of Baseline Characteristics between Study Groups

We identified 425,583 children who were eligible for this study (Figure 1). There were 139,659 (32.8%), 255,670 (60.1%), and 30,254 (7.1%) children in the low milk group, reference group, and high milk group, respectively. More than half of Korean children were reported to drink 200–499 mL milk per day. Even in the low milk group, 35,405 (8.3%) children did not drink milk at all.

Table 1 summarizes the baseline sociodemographic characteristics of the three groups. The distributions of sex, birth residence, income quintile, birth year, prematurity, birth weight, type of milk feeding from the age of 4–6 months, and amounts of SCB from the age of 18–24 months were similar across the three groups.

### 3.2. Primary Outcome: The Association between Milk Consumption and Obesity in Children

We examined the association between milk consumption and obesity using modified Poisson regression analysis. In the total cohort, we included subjects whose BMI was recorded at least once among the 5th (42–48 months), 6th (54–60 months), and 7th (66–71 months) rounds of NHSPIC (*n* = 377,592). Among the recorded BMI of the three rounds, the BMI of the last round of NHSPIC was analyzed. Obesity was defined as BMI z-score of ≥1.64. In the low milk group, 10,606 (8.54%) children were found to be obese; in the reference group, 23,138 (10.19%) children were obese; and in the high milk group, 3270 (12.39%) children were determined to be obese (*p* < 0.001).

Table 2 shows the adiposity outcomes at the age of 42–72 months based on the quantity of milk intake at the age of 30–36 months. After adjusting for sex, birth residence, income quintile, birth year, prematurity, birth weight, type of milk feeding from the age of 4 to 6 months, amounts of SCB from the age of 18 to 24 months (Table 1), and obesity at the 4th round of NHSPIC, a positive correlation was observed between milk consumption and obesity. In the adjusted analysis, the aRRs for obesity occurrence were 0.856 (95% CI, 0.835–0.878) for the low milk group and 1.120 (95% CI, 1.077–1.165) for the high milk group. Compared with the reference group, the risk of obesity was significantly greater for children who drank ≥500 mL milk per day.

### 3.3. Secondary Outcome: The Association between Milk Consumption and IDA in Children

We next analyzed the association between milk consumption and IDA in the total cohort. As per our findings, we observed that 1.86% of children in the reference and low milk groups and 2.06% of children in the high milk group were diagnosed with IDA and prescribed iron, respectively (*p* = 0.047). After adjusting for sociodemographic characteristics (Table 1), the risk of IDA was significantly increased in children who drank ≥500 mL milk per day (aRR, 1.079; 95% CI, 1.000–1.176) compared with the reference group (Table 3). No statistical significance was detected between the low milk group and the reference group.

## 4. Discussion

This study was conducted to demonstrate the current status of daily milk consumption in children aged 30–36 months in Korea and to identify its association with health conditions such as obesity and IDA in preschool-aged children. We observed that 60.1% of Korean children drank 200–499 mL milk per day, similar to the recommendations, whereas 7.1% drank >500 mL milk per day. Our results confirmed that children aged between 30 and 36 months who consumed >500 mL milk per day were at an increased risk of obesity at the age of 42–72 months, controlling for various confounding variables. The analysis with regard to overweight (BMI z-score ≥ 1.03) also revealed the same significant trend as that of obesity (Supplementary Table S2).

Our finding was consistent with that of previous studies. A US study of the 1999–2004 National Health and Nutrition Examination Surveys suggested consistent positive associations between milk intake and BMI among children aged 2–4 years old [19]. Another longitudinal cohort study of 12,829 US children aged 9–14 years reported that children who drank more than three servings of milk daily had an increased BMI [23]. Mark et al. [20] demonstrated that the volume of milk consumed was related to higher weight status and taller stature. Several potential mechanisms have been speculated in previous studies to explain the relationship between milk intake and obesity. Two studies suggested that the surplus energy provided by milk was stored as fat, thus leading to weight gain by increasing the total calorie consumption [21,23]. Another study speculated that milk has a unique biological effect of weight gain, considering that cow’s milk induces rapid growth in calves in terms of skeletal size and body weight [19]. There is also the ‘early protein hypothesis’ that high protein intake in infancy increases the risk of obesity [38,39]. A systematic literature review study of 34 articles concluded that high protein intake during infancy and early childhood was associated with an increased risk of obesity later in life [40]. However, there is no clear information about which components of milk have the potential to influence BMI; thus, further research is needed on this issue. However, it is true that milk is the only food that is actually produced by mammals for the purpose of childhood consumption [19].

This study was significant, considering that the analyzed age of obesity was 42–72 months, which is the AR period. BMI increases rapidly during the first year of life and then gradually decreases, reaching a minimum at 4–7 years of age, before increasing again during adolescence [31]. That point of minimal BMI has been termed the AR, and early AR has been identified as an indicator predicting later obesity [32]. Similarly, it is conceivable that becoming obese at the age of 4–7 years increases the risk of obesity in adolescence or early adulthood. Therefore, if a large amount of milk consumption is associated with a risk of obesity during this period, this also indicates the possibility of continuing obesity in the future.

We also analyzed additional outcomes about the relationship between milk intake and the risk of IDA by obtaining more information about milk and nutritional status in children. As per our results, it was determined that the risk of IDA was significantly increased in children who drank ≥500 mL milk per day compared with the reference group, and this finding was consistent with previous studies. Iron deficiency and IDA are important health issues in young children, given that they are associated with a negative impact on neurodevelopment [41]. Although the importance and prevention of IDA have focused on the first 12 months of life, it is also necessary to focus on toddlers, as the prevalence of IDA between 1 and 3 years is 15%, compared to 3% among those aged 1–2 years [28,29,42]. Some of the previously suggested mechanisms regarding milk intake and the occurrence of IDA include the low iron content of cow’s milk, low iron bioavailability for absorption, inhibition of iron absorption due to high amounts of the calcium and casein provided by milk, and occult intestinal blood loss [3]. Especially at the age of toddlers, the preference for food and beverages increases, often resulting in the refusal of healthy iron-rich foods and replacement with significant amounts of milk and fruit juices [41]. For this reason, the Centers for Disease Control and Prevention in the USA limited milk consumption to <24 oz per day in the second year of life [43], with some clinicians suggesting a stricter limit of 16 oz (approximately 473 mL) per day [29]. Furthermore, our experience in clinic shows that the majority of children who consume a large amount of milk at the age of 30–36 months are still dependent on bottle-feeding. There are several studies supporting associations between bottle-feeding beyond 15 to 18 months of age, excessive milk intake, and iron deficiency [24,25,26].

The strength of our study is the large sample size that makes our results reliable and generalizable. The study sample included all children born in Korea from 2008 to 2009, making it a nationally representative sample. However, we recognize some limitations of this study. First, as misreporting is common in diet surveys [1], recall bias may exist, as dietary information was self-reported, and it can be difficult for parents to accurately estimate children’s milk intake. Second, the questionnaire of the NHSPIC program did not include the consumption of other dairy products and did not distinguish the type of milk, such as whole, low-fat, or skim milk. However, previous studies have shown no relationship between the type of milk consumed and body weight status [18,44], or the association between BMI and dairy products was less consistent than that of milk [19]. Third, we could not adjust for all confounding factors, including physical activity time and energy intake from other foods. Instead, we attempted to adjust for as many confounding factors that could affect obesity as possible, such as prematurity, birth weight, socioeconomic status, feeding type from the age of 4 to 6 months, daily SCB consumption between the age of 18 and 24 months, and body weight status at the age of 30–36 months. In particular, one study demonstrated that children aged 24–47.9 months were more likely to consume SCB with each meal than children of other age groups [2], and much convincing evidence already exists indicating that SCB consumption increases the risk of overweight and obesity [45,46,47,48]. In our study, a meaningful result could be that the statistical significance was maintained even after adjusting for daily SCB consumption. Moreover, a significant outcome after adjustment for obesity at the 4th round of NHSPIC could be considered as an important factor. Fourth, the definition of IDA was based on the diagnosis of ICD-10 code along with the prescription of iron supplements. Because the diagnosis or prescription was not made by one person based on the same criteria, this value seems questionable. Further research is required to address these factors.

## 5. Conclusions

This national study demonstrated the current status of daily milk consumption in children aged 30–36 months in Korea as follows: 24.5% of children drank <200 mL milk per day, 60.1% of children drank 200–499 mL milk per day, 7.1% of children drank >500 mL milk per day, and 8.3% of children did not drink milk at all. Consumption of a large amount of milk of ≥500 mL per day at the age of 30–36 months was associated with an increased risk of obesity at the age of 42–72 months and IDA after the age of 30 months. These results indicate that the parents or guardians of children who consume >500 mL milk per day need education with regard to obesity prevention and consuming not only dairy products but also foods rich in iron or other essential nutrients that have low levels in milk. We believe that this study is significant, as it may provide partial evidence for dietary guidelines for milk consumption in children that are conducive to health.

## Figures and Tables

**Table 1:** Baseline sociodemographic characteristics of children in the low milk, reference, and high milk group.

Parameters	*n* (%)			
	Total Cohort (*n* = 425,583)	Low Milk Group ^a^ (*n* = 139,659)	Reference Group ^b^ (*n* = 255,670)	High Milk Group ^c^ (*n* = 30,254)
Sex				
Male	214,894 (50.5)	67,396 (48.3)	130,930 (51.2)	16,568 (54.8)
Female	210,689 (49.5)	72,263 (51.7)	124,740 (48.8)	13,686 (45.2)
Birth residence ^d^				
Seoul	106,558 (25.3)	36,840 (26.6)	62,124 (24.5)	7594 (25.4)
Metropolitan	96,609 (22.9)	29,145 (21.1)	60,481 (23.9)	6983 (23.4)
City	167,318 (39.7)	55,173 (39.9)	100,470 (39.7)	11,675 (39.1)
Rural	51,124 (12.1)	17,231 (12.5)	30,263 (11.9)	3630 (12.1)
Income quintile ^e^				
1 (Lowest)	33,429 (8.2)	10,294 (7.7)	20,421 (8.3)	2714 (9.3)
2	61,501 (15.0)	19,274 (14.3)	37,363 (15.2)	4864 (16.7)
3	112,116 (27.3)	35,996 (26.8)	68,201 (27.7)	7919 (27.2)
4	133,834 (32.6)	44,993 (33.5)	79,946 (32.4)	8895 (30.6)
5 (Highest)	69,206 (16.9)	23,935 (17.8)	40,570 (16.5)	4701 (16.2)
Birth year of child				
2008	203,375 (47.8)	65,945 (47.2)	122,655 (48.0)	14,775 (48.8)
2009	222,208 (52.2)	73,714 (52.8)	133,015 (52.0)	15,479 (51.2)
Prematurity ^f^				
No	408,847 (96.1)	134,384 (96.3)	245,508 (96.1)	28,955 (95.8)
Yes	16,445 (3.9)	5201 (3.7)	9972 (3.9)	1272 (4.2)
Birth weight, mean(SD), kg				
	3.22 (0.41)	3.22 (0.41)	3.22 (0.41)	3.21 (0.42)
Type of milk feeding from age 4 to 6 months ^g^				
Breast	115,785 (45.5)	39,945 (47.3)	69,906 (45.6)	5934 (35.3)
Bottle	87,885 (34.5)	27,858 (33.0)	52,681 (34.3)	7346 (43.7)
Mixed ^h^	50,132 (19.7)	16,285 (19.3)	30,383 (19.8)	3464 (20.6)
Special formula	889 (0.3)	312 (0.4)	494 (0.3)	83 (0.5)
Amounts of SCB from age 18 to 24 months ^i^				
<200 mL/d	263,760 (93.3)	87,068 (93.7)	159,808 (93.3)	16,884 (91.8)
200 to 499 mL/d	17,315 (6.1)	5423 (5.8)	10,607 (6.2)	1285 (7.0)
≥500 mL/d	1531 (0.5)	416 (0.5)	884 (0.5)	231 (1.3)

Data are presented as number (percent) or mean (SD). Abbreviation: SCB, sugar-containing beverages. ^a^ The group of children who drink <200 mL milk per day. ^b^ The group of children who drink 200–499 mL milk per day. ^c^ The group of children who drink ≥500 mL milk per day. ^d^ Residential status was classified as Seoul, metropolitan, urban, or rural. Metropolitan areas were defined as five metropolitan cities (Busan, Incheon, Gwangju, Daejeon, and Ulsan), urban areas as cities, and rural areas as non-city areas. Of all the participants, information was missing for 1270 in low milk group, 2332 in reference group, and 372 in high milk group. ^e^ Income was categorized into quintiles of average neighborhood income on the index date. Of all the participants, information was missing for 5167 in low milk group, 9169 in reference group, and 1161 in high milk group. ^f^ Of all the participants, information was missing for 74 in low milk group, 190 in reference group, and 27 in high milk group. ^g^ Question in the first round of the NHSPIC program: “What do you usually feed your child with?”. Of all the participants, information was missing for 55,259 in low milk group, 102,206 in reference group, and 13,427 in high milk group. ^h^ Feeding with breast milk, animal milk, and/or formula milk. ^i^ Parents answered the following question in the third round of the NHSPIC program: “How much fruit juices or sugary beverages does your child drink per day?” with the following possible answers: (1) <200 mL, (2) 200–499 mL, and (3) ≥500 mL. Of all the participants, information was missing for 46,752 in low milk group, 84,371 in reference group, and 11,854 in high milk group.

**Table 2:** The association between the milk consumption and obesity in children.

Milk Consumption (mL/d)	*n* = 377,592 ^a^		RR (95% CI)	
	Subjects, *n*	Obesity ^b^, *n* (%)	Unadjusted	Adjusted ^c^
Low Milk Group ^d^	124,236	10,606 (8.54)	**0.820** **(0.805 to 0.835)**	**0.856** **(0.835 to 0.878)**
Reference Group ^e^	226,964	23,138 (10.19)	Ref	Ref
High Milk Group ^f^	26,392	3270 (12.39)	**1.249** **(1.214 to 1.284)**	**1.120** **(1.077 to 1.165)**

Abbreviation: RR, relative risk; CI, confidence interval; BMI, body mass index. ^a^ Subjects who recorded BMI at least once between the 5th to 7th rounds of NHSPIC were included. ^b^ Obesity was defined as BMI z-score ≥ 1.64, based on the BMI recorded at the last round of the 5th to 7th NHSPIC. ^c^ Adjusted for sociodemographic characteristics (Table 1), and obesity at the 4th round of NHSPIC, as recorded in the database. ^d^ The group of children who drink <200 mL milk per day. ^e^ The group of children who drink 200–499 mL milk per day. ^f^ The group of children who drink ≥500 mL milk per day. Bold values indicate *p* < 0.05.

**Table 3:** The association between the milk consumption and IDA^a^ in children.

Milk Consumption (mL/d)	*n* = 455,160		RR (95% CI)	
	Subjects, *n*	IDA, *n* (%)	Unadjusted	Adjusted ^b^
Low Milk Group ^c^	139,659	2593 (1.86)	1.001 (0.954 to 1.050)	1.016 (0.967 to 1.067)
Reference Group ^d^	255,670	4744 (1.86)	Ref	Ref
High Milk Group ^e^	30,254	622 (2.06)	**1.108** **(1.019 to 1.205)**	**1.079** **(1.000 to 1.176)**

Abbreviations: RR, relative risk; CI, confidence interval; BMI, body mass index. ^a^ IDA was defined with at least one diagnosis of ICD-10 code D50.8 or, D50.9 along with prescription of iron using drug classification codes 322, at least once from the 4th round of NHSPIC to December 2017. ^b^ Adjusted for sociodemographic characteristics (Table 1), as recorded in the database. ^c^ The group of children who drink <200 mL milk per day. ^d^ The group of children who drink 200–499 mL milk per day. ^e^ The group of children who drink ≥500 mL milk per day. Bold values indicate *p* < 0.05.

## Data Availability

This study was based on the National Health Claims Database (NHIS-2019-1-560) established by the NHIS of the Republic of Korea. Applications for using NHIS data are reviewed by the Inquiry Committee of Research Support; if the application is approved, raw data is provided to the applicant for a fee. We cannot provide access to the data, analytic methods, and research materials to other researchers because of the intellectual property rights of this database, which are owned by the National Health Insurance Corporation. However, investigators who wish to reproduce our results or replicate the procedure can use the database, which is open for research purposes (https://nhiss.nhis.or.kr/ accessed on 1 October 2021).

## Supplementary Materials

The following are available online at https://www.mdpi.com/article/10.3390/nu13103494/s1. Table S1: Drug classification codes 322 in NHIS database. Table S2: The association between the milk consumption and overweight in children.

## References

[1] Guo Q., Wang B., Cao S., Jia C., Yu X., Zhao L., Dellarco M., Duan X. (2020). Association between milk intake and childhood growth: Results from a nationwide cross-sectional survey. Int. J. Obes..

[2] Kay M.C., Welker E.B., Jacquier E.F., Story M. (2018). Beverage consumption patterns among infants and young children (0–47.9 months): Data from the Feeding Infants and Toddlers Study, 2016. Nutrients.

[3] Agostoni C., Turck D. (2011). Is cow’s milk harmful to a child’s health?. J. Pediatr. Gastroenterol. Nutr..

[4] Lee K.W., Cho W. (2017). The consumption of dairy products is associated with reduced risks of obesity and metabolic syndrome in Korean women but not in men. Nutrients.

[5] US Department of Health and Human Services, US Department of Agriculture 2015–2020 Dietary Guidelines for Americans. http://www.health.gov/DietaryGuidelines.

[6] Chinese Nutrition Society (2016). Chinese Dietary Guidelines.

[7] Korean Ministry of Health and Welfare, The Korean Nutrition Society (2020). 2020 Dietary Reference Intakes for Koreans: Energy and Macronutrients.

[8] Holmes M.D., Pollak M.N., Willett W.C., Hankinson S.E. (2002). Dietary correlates of plasma insulin-like growth factor I and insulin-like growth factor binding protein 3 concentrations. Cancer Epidemiol. Biomark. Prev..

[9] Kim S.H., Kim W.K., Kang M.-H. (2013). Effect of milk and milk products consumption on physical growth and bone mineral density in Korean adolescents. Nutr. Res. Pract..

[10] Marshall T.A., Curtis A.M., Cavanaugh J.E., Warren J.J., Levy S.M. (2018). Higher longitudinal milk intakes are associated with increased height in a birth cohort followed for 17 years. J. Nutr..

[11] Feskanich D., Bischoff-Ferrari H.A., Frazier A.L., Willett W.C. (2014). Milk consumption during teenage years and risk of hip fractures in older adults. JAMA Pediatr..

[12] Berkey C.S., Colditz G.A., Rockett H.R., Frazier A.L., Willett W.C. (2009). Dairy consumption and female height growth: Prospective cohort study. Cancer Epidemiol. Biomark. Prev..

[13] de Beer H. (2012). Dairy products and physical stature: A systematic review and meta-analysis of controlled trials. Econ. Hum. Biol..

[14] Wiley A.S. (2005). Does milk make children grow? Relationships between milk consumption and height in NHANES 1999–2002. Am. J. Hum. Biol..

[15] Spence L.A., Cifelli C.J., Miller G.D. (2011). The role of dairy products in healthy weight and body composition in children and adolescents. Curr. Nutr. Food Sci..

[16] Abreu S., Santos R., Moreira C., Vale S., Santos P.C., Soares-Miranda L., Marques A.I., Mota J., Moreira P. (2012). Association between dairy product intake and abdominal obesity in Azorean adolescents. Eur. J. Clin. Nutr..

[17] Beck A.L., Heyman M., Chao C., Wojcicki J. (2017). Full fat milk consumption protects against severe childhood obesity in Latinos. Prev. Med. Rep..

[18] Huh S.Y., Rifas-Shiman S.L., Rich-Edwards J.W., Taveras E.M., Gillman M.W. (2010). Prospective association between milk intake and adiposity in preschool-aged children. J. Am. Diet. Assoc..

[19] Wiley A.S. (2010). Dairy and milk consumption and child growth: Is BMI involved? An analysis of NHANES 1999–2004. Am. J. Hum. Biol..

[20] DeBoer M.D., Agard H.E., Scharf R.J. (2015). Milk intake, height and body mass index in preschool children. Arch. Dis. Child..

[21] O’Connor T.M., Yang S.J., Nicklas T.A. (2006). Beverage intake among preschool children and its effect on weight status. Pediatrics.

[22] Kral T.V., Stunkard A.J., Berkowitz R.I., Stallings V.A., Moores R.H., Faith M.S. (2008). Beverage consumption born at different risk of patterns of children obesity. Obesity.

[23] Berkey C.S., Rockett H.R., Willett W.C., Colditz G.A. (2005). Milk, dairy fat, dietary calcium, and weight gain: A longitudinal study of adolescents. Arch. Pediatr. Adolesc. Med..

[24] Safer D.L., Bryson S., Agras W.S., Hammer L.D. (2001). Prolonged bottle feeding in a cohort of children: Does it affect caloric intake and dietary composition?. Clin. Pediatr. (Phila).

[25] Lampe J.B., Velez N. (1997). The effect of prolonged bottle feeding on cow’s milk intake and iron stores at 18 months of age. Clin. Pediatr. (Phila).

[26] Maguire J.L., Birken C.S., Jacobson S., Peer M., Taylor C., Khambalia A., Mekky M., Thorpe K.E., Parkin P. (2010). Office-based intervention to reduce bottle use among toddlers: TARGet Kids! Pragmatic, randomized trial. Pediatrics.

[27] Ziegler E.E. (2011). Consumption of cow’s milk as a cause of iron deficiency in infants and toddlers. Nutr. Rev..

[28] Looker A.C., Dallman P.R., Carroll M.D., Gunter E.W., Johnson C.L. (1997). Prevalence of iron deficiency in the United States. JAMA.

[29] Kazal L.A. (2002). Prevention of iron deficiency in infants and toddlers. Am. Fam. Physician.

[30] Kim J.H., Lee J.E., Shim S.M., Ha E.K., Yon D.K., Kim O.H., Baek J.H., Koh H.Y., Chae K.Y., Lee S.W. (2021). Cohort profile: National Investigation of Birth Cohort in Korea study 2008 (NICKs-2008). Clin. Exp. Pediatr..

[31] Whitaker R.C., Pepe M.S., Wright J.A., Seidel K.D., Dietz W.H. (1998). Early adiposity rebound and the risk of adult obesity. Pediatrics.

[32] Rolland-Cachera M., Deheeger M., Maillot M., Bellisle F. (2006). Early adiposity rebound: Causes and consequences for obesity in children and adults. Int. J. Obes. (Lond.).

[33] Centers for Disease Control and Prevention Defining Childhood Weight Status BMI for Children and Teens. https://www.cdc.gov/obesity/childhood/defining.html.

[34] Gallagher D.A. A Guide to Methods for Assessing Childhood Obesity. https://www.nccor.org/tools-assessingobesity.

[35] Kwon Y., Jeong S.J. (2019). Association between Body Mass Index and Hepatitis B antibody seropositivity in children. Korean J. Pediatr..

[36] Kwon Y., Kim J.H., Ha E.K., Jee H.M., Baek H.S., Han M.Y., Jeong S.J. (2020). Serum YKL-40 Levels Are Associated with the Atherogenic Index of Plasma in Children. Mediat. Inflamm..

[37] Lee J., Kim J.H. (2021). Endocrine comorbidities of pediatric obesity. Clin. Exp. Pediatr..

[38] Rolland-Cachera M.F., Deheeger M., Akrout M., Bellisle F. (1995). Influence of macronutrients on adiposity development: A follow up study of nutrition and growth from 10 months to 8 years of age. Int. J. Obes. Relat. Metab. Disord..

[39] Xu S., Xue Y. (2016). Protein intake and obesity in young adolescents. Exp. Ther. Med..

[40] Hörnell A., Lagström H., Lande B., Thorsdottir I. (2013). Protein intake from 0 to 18 years of age and its relation to health: A systematic literature review for the 5th Nordic Nutrition Recommendations. Food Nutr. Res..

[41] Bondi S.A., Lieuw K. (2009). Excessive Cow’s Milk Consumption and Iron Deficiency in Toddlers: Two Unusual Presentations and Review. Infant Child. Adolesc. Nutr..

[42] Eden A.N., Mir M.A. (1997). Iron deficiency in 1-to 3-year-old children: A pediatric failure?. Arch. Pediatr. Adolesc. Med..

[43] Centers for Disease Control and Prevention (1998). Recommendations to prevent and control iron deficiency in the United States. MMWR Recomm. Rep..

[44] Scharf R.J., Demmer R.T., DeBoer M.D. (2013). Longitudinal evaluation of milk type consumed and weight status in preschoolers. Arch. Dis. Child..

[45] Borges M.C., Louzada M.L., de Sá T.H., Laverty A.A., Parra D.C., Garzillo J.M., Monteiro C.A., Millett C. (2017). Artificially sweetened beverages and the response to the global obesity crisis. PLoS Med..

[46] Imamura F., O’Connor L., Ye Z., Mursu J., Hayashino Y., Bhupathiraju S.N., Forouhi N.G. (2015). Consumption of sugar sweetened beverages, artificially sweetened beverages, and fruit juice and incidence of type 2 diabetes: Systematic review, meta-analysis, and estimation of population attributable fraction. BMJ.

[47] Te Morenga L., Mallard S., Mann J. (2013). Dietary sugars and body weight: Systematic review and meta-analyses of randomised controlled trials and cohort studies. BMJ.

[48] Hu F.B. (2013). Resolved: There is sufficient scientific evidence that decreasing sugar-sweetened beverage consumption will reduce the prevalence of obesity and obesity-related diseases. Obes. Rev..

