# Supplementary material for: Is High Milk Intake Good for Children’s Health? A National Population-Based Observational Cohort Study
Source: Nutrients. 2021 Oct 2;13(10):3494. doi: 10.3390/nu13103494 (PMC8541527; doi:10.3390/nu13103494)
Supplement: Supplementary file 1 [file nutrients-13-03494-s001.zip › nutrients-1341715-supplementary.pdf]

**Supplementary Table S1.** Drug classification codes 322 in NHIS database

| | | |
|---|---|---|
| 322 | Ferric hydroxide polymaltose complex | as Fe III 0.1g(6.667mg/mL) |
| | | as Fe III 0.1g(10mg/mL) |
| | | as Fe III 0.1g(20mg/mL) |
| | | as Fe III 1.5g(50mg/mL) |
| | | as Fe III 2.25g(50mg/mL) |
| | | as Fe III 2.5g(50mg/mL) |
| | | as Fe III 3g(50mg/mL) |
| | | as Fe III 5g(50mg/mL) |
| | | as Fe 0.1g |
| | Iron proteinsuccinylate | as Fe 20mg |
| | | as Fe III 40mg(2.667mg/mL) |
| | Polysaccharide iron complex | as Fe 50mg |
| | | as Fe 0.15g |
| | | as Fe 0.1g(5mg/mL) |
| | Sodium ferric gluconate complex | as Fe III 62.5mg |
| | | as Fe III 62.5mg(6.25mg/mL) |
| | | as Fe III 62.5mg(7.8125mg/mL) |
| | Folic acid | 0.35mg |
| | Ferrous sulfate dried | as Fe II 80mg |
| | Carbonyl iron | 45mg |
| | Iron acetyl transferrin | as Fe 40mg |
| | | as Fe 40mg(4mg/mL) |
| | Ferritinic iron | 40mg(4mg/mL) |
| | Calcium folinate | 0.3mg(37.5μg/mL) |
| | Iron dextran | as iron 0.1g(50mg/mL) |
| | Iron hydoxide sucrose complex | as Fe 0.1g(20mg/mL) |
| | | as Fe 0.2g(20mg/mL) |

Abbreviation: NHIS, National Health Insurance System.

**Supplementary Table S2.** The association between the milk consumption and overweight in children

| **Milk Consumption (mL/d)** | **N=377,592[a]** | | **RR (95% CI)** | |
|---|---|---|---|---|
| | **Subjects, N** | **Overweight[b], N (%)** | **Unadjusted** | **Adjusted[c]** |
| Low Milk Group[d] | 124,236 | 28,927 (23.28) | **0.851 (0.842 to 0.860)** | **0.904 (0.892 to 0.916)** |
| Reference Group[e] | 226,964 | 60,700 (26.74) | Ref | Ref |
| High Milk Group[f] | 26,392 | 7,931 (30.05) | **1.151 (1.133 to 1.170)** | **1.120 (1.010 to 1.056)** |

Abbreviation: RR, relative risk; CI, confidence interval; BMI, body mass index.

[a] Subjects who recorded BMI at least once among the 5th to 7th rounds of NHSPIC were included.

[b] Obesity was defined as BMI for age z score ≥ 1.03, based on the BMI recorded at the last round among the 5th to 7th NHSPIC.

[c] Adjusted for sociodemographic characteristics(Table 1), and obesity at the 4th round of NHSPIC, as recorded in the database.

[d] The group of children who drink <200 mL milk per day.

[e] The group of children who drink 200–499 mL milk per day.

[f] The group of children who drink ≥500 mL milk per day.

Bold values indicate $p < 0.05$
